# Content Analysis of the Construction of Self and Others in Women with Bulimia Nervosa

**DOI:** 10.3389/fpsyg.2017.00480

**Published:** 2017-04-06

**Authors:** Gloria Dada, Sheila Izu, Claudia Montebruno, Antoni Grau, Guillem Feixas

**Affiliations:** ^1^Werklund School of Education – Counselling Psychology, University of Calgary, CalgaryAB, Canada; ^2^Clinical Psychology, Centro ArboretumSan Salvador, El Salvador; ^3^Department of Personality, Assessment and Psychological Treatment, Universitat de BarcelonaBarcelona, Spain; ^4^Centro AidaSantiago, Chile; ^5^Institut de Trastorns AlimentarisBarcelona, Spain

**Keywords:** bulimia nervosa, RepGrid, personal construct, content analysis, eating disorders

## Abstract

The purpose of this study was to explore the content of personal constructs in people diagnosed with bulimia nervosa (BN). We expected to find differences in the predominant content of the construct systems between women with and without BN. We analyzed the constructs elicited using the repertory grid technique from 120 women aged between 18 to 45 years, divided into two groups: a clinical group of women diagnosed with bulimia (*n* = 62) and a control group of university students (*n* = 58). The constructs were categorized using the Classification System for Personal Constructs (CSPC), composed of six themes which are broken down into 45 categories. For this study, a new area called “Physical” was included, and it consists of three categories. The results indicated that women diagnosed with bulimia used significantly more constructs related to the body, while the control group used more constructs from the personal area. In addition, the congruent constructs from the clinical sample were predominantly moral, or related to values and interests, while discrepant constructs were personal and physical. The findings provide evidence for the clinical use of the CSPC as an instrument for exploring the content of personal meaning systems. Understanding the patient’s personal constructions about herself and others is useful for treatment. Moreover, it is important for clinicians to explore the content of constructs related to symptomatic areas, which could be hindering change, and focus on them to facilitate improvement.

## Introduction

As reflected in the DSM-5 (American Psychiatric Association [APA], 2013), diagnostic criteria for the eating disorders (ED) include the undue influence of body shape and weight on self-evaluation. Weight-based self-evaluation is a core feature of psychopathology common to Anorexia Nervosa (AN), Bulimia Nervosa (BN), Binge Eating Disorder (BED) and other cases of EDs (e.g., Fairburn et al., 2003; Fairburn, 2008; Grilo et al., 2013). Furthermore, this evaluation of the self, based on weight and shape, seems to be resistant to treatment and it is considered a maintaining factor and a predictor of relapse (Grilo et al., 2013; Trottier et al., 2013). In fact, evaluating self and self-worth mostly –or even exclusively- based on body features, combined with a perfectionistic ideal of shape and weight, is a vulnerability factor for low self-esteem (Lampard and Sharbanee, 2015).

The value and adequacy attributed to the self, usually referred to as self-esteem, is one of the most widely studied factors relating to EDs from different theoretical perspectives (e.g., Garner and Bemis, 1982; Button et al., 1996; Fairburn et al., 2003; Jacobi et al., 2004; Welch and Ghaderi, 2013), though not an exclusive factor for these disorders. More than 15 years ago, Bruch (1962, 1978) emphasized the feeling of inadequacy as an underlying psychological characteristic of patients with EDs, indicating lack of a clear and cohesive sense of self. More specifically, it has been remarked in the clinical and research literature that patients with BN lack a clearly defined sense of self and, therefore, they use their physical bodies as a means of self-definition and regulation (e.g., Schupak-Neuberg and Nemeroff, 1993). Eating related symptoms (such as restrained eating, binge eating, or purging) could be understood as attempts to improve the sense of self-worth and/or ease the negative emotions related to a negative self-esteem (Fairburn, 2008).

Even though a negative self-concept and impoverished identity have been widely related to EDs in general, and to BN in particular (Stein and Corte, 2007), previous research has operationalized these aspects of self-construing in different ways. Watson and Watts (2001) emphasized that the sense of self-worth is based on the similarity (or discrepancy) between the perception of the current self and the ideal self, in terms of those attributes deemed to be important for the person. This approach supports the use of theories that promote systematic assessment of the personal (and subjective) views of patients.

This present study is part of the *Multicentre Dilemma Project*^1^, a program of research focused on the role of cognitive conflicts in health, which is inspired by Kelly’s Personal Construct Theory (PCT; see Walker and Winter, 2007). PCT is grounded in a constructivist epistemology (Neimeyer and Mahoney, 1995; Botella and Feixas, 1998; Feixas and Villegas, 2000; Winter and Reed, 2015), according to which reality can only be accessed through interpretative dimensions erected by the subject. Kelly (1955/1991) suggested that human beings live and understand experience like scientists who build [informal] hypotheses to interpret, organize, and anticipate their experiences. Self, others, and reality are construed using a set of distinctions, known as *personal constructs*, created uniquely and personally from previous experience. They are usually represented as bipolar dimensions of meaning (such as “weak-strong” or “fat-thin”). By doing this, an interdependent, hierarchical, and complex meaning network is built, by which a person can understand and anticipate experience. In this network, some lower level constructs could be directly related to others of higher significance for the individual.

There are several techniques to explore someone’s personal constructs (e.g., self-characterization, laddering), but the most widely used is the Repertory Grid Technique (RGT), devised to reveal the dimensions and structure of the personal construct system (e.g., Fransella et al., 2004; Saúl et al., 2012). This semi-structured interview aims to identify the constructs by which a person idiosyncratically gives sense and meaning to experiences and organizes his or her world.

Consistent with the constructivist epistemology, RGT is useful for the assessment of perceived self-adequacy, based on those characteristics that are relevant to the person, and not in relation to items derived from theoretical constructs, or to the general desirability of certain features. RGT measures self-discrepancies based on Euclidean distances between current self and ideal self. Besides this general index of self-adequacy, it also provides information about self-discrepancies in each one of the constructs, taken individually. A construct is considered *congruent* if the position of current self and ideal self are similar, indicating that the person is, in that respect, the way he/she would like to be. On the other hand, if the current self is rated at one pole of the construct and the ideal self is positioned on the opposite pole; the construct is considered *discrepant*, indicating that this is an aspect in which the person wants to change.

The RGT has been applied to study a broad variety of areas of clinical (e.g., depression, schizophrenia, agoraphobia, self-injury) and health (irritable bowel syndrome, chronic pain, fibromyalgia, cancer) psychology. EDs have also been studied by PCT researchers using the RGT (e.g., Fransella and Crisp, 1970, 1979; Neimeyer and Khouzam, 1985; Button, 1993; Izu, 2007; Feixas et al., 2010; Dada et al., 2012). These studies showed that structural relationships among constructs and cognitive conflicts assessed with the RGT are useful for understanding the construing process of patients with EDs.

Within the *Multicentre Dilemma Project*, previous studies have focused on cognitive conflicts in EDs, with particular attention to Implicative Dilemmas (ID). An ID can be understood as a strong relationship with a construct based on which the person wishes to change (discrepant construct) and a construct on which changing is not desirable (congruent construct). Due to this association, change in the desired direction on the discrepant construct implies change in the undesired direction on the congruent construct (Feixas and Saúl, 2004). Previous studies found that IDs were more frequent in the grids of ED patients compared to women from non-clinical samples (Izu, 2007; Feixas et al., 2010; Dada et al., 2012; Dada, 2014). Nevertheless, the content of constructs involved in those dilemmas has received less attention.

Even though there is a considerable amount of research on the structural characteristics of construing, studies on the content of personal constructs is scarce. Landfield (1971) proposed a general classification system to categorize personal constructs. This system, however, had several methodological disadvantages such as not being exhaustive (Feixas, 1988). Feixas et al. (2002) presented the Classification System for Personal Constructs (CSPC) comprising 45 exclusive and comprehensive categories. This system is structured according to homogeneous levels of abstraction, and is based solely on the content of the constructs.

The CSPC has been used in previous research to analyze the patterns of construct content related to different clinical samples, such as patients with depression (Montesano et al., 2009; Feixas et al., 2014), women with fibromyalgia (Compañ et al., 2011) and victims of sexual abuse (Harter et al., 2004). To the extent of our knowledge, the content of personal constructs from BN patients has not been studied yet.

The purpose of this research is to analyze the cognitive content of women diagnosed with BN, and to compare it to that of a non-clinical sample in order to advance knowledge of their personal construct systems. Also, the content of the constructs involved in self-construction and in cognitive conflicts in patients with BN will be specifically examined.

## Materials and Methods

### Participants

The clinical group was comprised of 62 women recruited in two cohorts, from two previous studies (Izu, 2007; Feixas et al., 2010). All patients were recruited within the first month of treatment at the *Institut de Trastorns Alimentaris* (Barcelona, Spain), a treatment facility specializing in eating disorders, either in full or partial hospitalization. They fulfilled diagnostic criteria for BN at admission for treatment, and when assessed for this study. Age of patients ranged between 18–45 years (*M* = 25.08; *SD* = 6.16).

The control group was formed of 58 women, either undergraduate or graduate students from the University of Barcelona. During the assessment interview, the questions from the section on Eating Disorders of the Structured Clinical Interview for DSM-IV Diagnosis (First et al., 1996) were used to screen participants in the control group. The age of participants composing the control group ranged between 18 and 45 years (*M* = 25.14; *SD* = 5.6) in age.

### Instruments and Measures

#### Repertory Grid Technique

This is a semi-structured interview, originally proposed by Kelly (1955/1991). It can be adapted for the purposes of each particular research or clinical interest (Fransella et al., 2004; Feixas and Cornejo, 2002), resulting in a variety of formats. The format used in this study is designed to explore the participants’ constructs about their interpersonal world. Initially, the interviewer elicited elements for inclusion in the grid, which included the self now, the “ideal self” (i.e., “How I would like to be”), and significant others identified by the participant (e.g., parents, siblings, friends, current and/or former partner, and a *persona non grata*). These elements were contrasted in dyads, eliciting constructs describing similarities and differences between elements. An opposite or “construct pole” was also described for each similarity or difference. Elicitation continued till the person was unable to generate new constructs. The mean number of constructs elicited for the grids of the student group was 32.04, (*SD* = 9.12) and did not differ significantly [*t*(119) = 0.50, *p* = 0.620] from that of the BN group (32.81, *SD* = 7.78).

Following elicitation of constructs, the interviewer asked the participant to rate each element on each of his or her constructs. For these ratings, a 7-point Likert scale was used, ranging from “very much like the construct’s left pole” to “very much like the construct’s right pole.” These ratings provided a data matrix for each participant, with columns representing important people (including “current self” and “ideal self”) in the interpersonal world of the interviewee, and rows representing her construct dimensions.

The grids were analyzed using the GRIDCOR 4.0 software (Feixas and Cornejo, 2002) to identify congruent constructs, discrepant constructs, and constructs involved in IDs.

A congruent construct is a construct in which the self and the ideal self are rated on the same pole, and the difference between the rating for these elements is lower or equal to one point. This similarity between ratings indicates a characteristic on which the respondent sees her actual self akin to how she would ideally like to be.

On the contrary, a discrepant construct indicates dissatisfaction, revealed by a discrepancy between the respondent’s self-representation and what she considers ideal. It is a construct on which the person wishes to change. This is evidenced by a difference of 4 or more points between the ratings given to the self and the ideal self, which are therefore positioned at opposite poles of the construct.

Implicative Dilemmas were identified using the GRIDCOR 4.0 Software, based on the correlations between congruent constructs and discrepant constructs (Feixas et al., 2000; Feixas and Saúl, 2004).

#### Classification System for Personal Constructs

This is a coding system developed by Feixas et al. (2002) designed to classify the content of personal constructs derived from the RGT and other constructivist assessment procedures. This qualitative analysis by categories is useful to explore the presence or absence of a variety of aspects of content in the personal construct systems of grid respondents.

The CSPC is composed of 45 exclusive and mutually exclusive categories classified into six thematic areas. These areas are hierarchically ordered to increase the reliability of the system by eliminating potential overlap among them: whenever a construct fits within two or more areas, it is coded in the area of the higher level. Categories belonging to the same area are considered as hierarchically equivalent.

The CSPC focuses on what Feixas (1988) regards as value constructs, which include the meanings or attributions about one’s and others’ psychological traits. Other characteristics such as physical attributes were excluded, because they were considered superficial or irrelevant distinctions. However, for the purpose of this study another area was added to the system, in order to classify those contents related to the construction of the body. This new area is called “Physical,” and it is composed of three categories: *body appearance*, *physical malaise*, and *attitude toward the body.* We provided examples of the constructs that could be codified in each one of the categories included in this area (see **Table 1**), using the same format as in all 45 categories from the CSPC.

**Table 1:** Categories included in the “Physical Area” included for the CSPC for the purposes of this study.

7A	BODY APPEARANCE	
	Thin	Fat
	Pretty	Ugly
7B	PHYSICAL MALAISE	
	In pain	Without pain
	Healthy	Ill
7C	ATTITUDE TOWARDS THE BODY	
	Worried about body	At ease with own body
	Takes care of own body	Does not care
	Obsessed with food (or weight, or body, or health)	Not obsessed with food (or weight, or body, or health)


Previous studies (Feixas et al., 2002; Montesano et al., 2009; Compañ et al., 2011; Feixas et al., 2014) have confirmed a high level of reliability of the CSPC. In effect, inter-rater agreement between judges encoding the same constructs separately and after discussing discrepancies in codings showed indices between 0.90 and 0.95 for both areas and categories. This level of reliability is quite satisfactory, more characterized of standardized scales in psychological testing than of a coding system.

### Procedure

This study was carried out with the approval of the Bioethics Commission from the University of Barcelona (Institutional Review Board IRB00003099), with written informed consent from all subjects. Students were recruited from undergraduate and graduate courses at the University. The clinical participants were selected from the registries of the above-mentioned treatment facility, based on the inclusion criteria for the study: women, over 18 years old, diagnosed with BN. A brief interview was conducted, to confirm the diagnosis.

Once identified, each participant was contacted by the researcher, informed about the purposes of the studies, and granted written informed consent.

An interviewer, trained in the use of the RGT, assessed those participants who fulfilled inclusion criteria.

All constructs from the participants’ grids were classified by two independent judges, using the CSPC. To avoid bias, judges were not informed about the study’s hypothesis. Both judges were postgraduate students who received training in the CSPC. Each judge separately analyzed a total of 3,777 constructs extracted from the RGT from all 120 participants. Once all constructs were categorized with the CSPC, both judges compared their classifications for every single construct, discussed their discrepancies and registered final agreement or disagreement. Those constructs on which no agreement was reached between judges were excluded from further analyses. Data were analyzed using the statistical package SPSS v. 20.

## Results

Inter-rater agreement was assessed using Cohen’s kappa statistic. Results indicated a very good agreement between judges before discussion (*K* = 0.813, *p* < 0.001), and after discussion (*K* = 0.968, *p* < 0.001). Therefore, the application of the CSPC can be considered as reliable.

A Chi-square test for independence indicated significant differences between groups regarding the distribution of constructs in the areas of content, χ^2^(7,*n* = 3,777) = 72.55, *p* < 0.001, φ = 0.14. In order to know which areas produced this difference between groups, standardized residuals (SR) were compared to the critical value of ±1.96 (α = 0.05). If the SR are greater than or equal to 1.96, or less than or equal to -1.96, then the observed frequency value is significantly different from the expected frequency (i.e., if the residual value is positive, then the group is over-represented, and if the value is negative, then the group is under-represented). As expected, constructs from the physical area were over-represented in the clinical sample. The non-clinical sample showed more constructs from the personal area than BN patients (see **Table 2**).

**Table 2:** Frequency and percentage of constructs, and standardized residuals by area and group.

		Moral	Emotional	Relational	Personal^∗^	Intellectual	Values	Physical^∗^
Non-clinical group (*n* = 58)	*f*	270	325	379	425	109	114	23
	*f_e_*	270.0	315.8	383.6	396.7	97.9	106.2	75.6
	%	7.1%	8.6%	10.0%	11.3%	2.9%	3.0%	0.6%
	SR	0.0	0.8	-0.4	2.2	1.5	1.0	-8.3
Clinical group (*n* = 62)	*f*	348	398	499	483	115	129	150
	*f_e_*	348.0	407.2	494.4	511.3	126.1	136.8	97.4
	%	9.2%	10.5%	13.2%	12.8%	3.0%	3.4%	4.0%
	SR	0.0	-0.8	0.4	-2.2	-1.5	-1.0	8.3
Total	*f*	618	723	878	908	224	243	173
	%	16.4%	19.1%	23.2%	24.0%	5.9%	6.4%	4.6%


*f, frequency of constructs; f_e_, expected frequency; SR, standardized residuals. ^∗^Areas in which frequency value is significantly different from the expected frequency.*

To analyze in more detail the differences between groups, a second analysis was performed to contrast the categories of constructs. Again, statistically significant differences between groups were found, χ^2^(48, *n* = 3776) = 137.586, *p* < 0.001, φ = 0.19.

As expected, constructs from the categories “body appearance” and “attitude toward the body” were overrepresented in BN patients compared to controls. On the other hand, participants of the non-clinical sample used more constructs from the “visceral-rational” category (See **Table 3**).

**Table 3:** Frequency of constructs and standardized residuals by area and category in BN patients and non-clinical controls.

Category		*f*		SR		Category		*f*		SR	
						
		NC	BN	NC	BN			NC	BN	NC	BN
1A	Good-bad	25	38	-0.6	0.6	3I	Trusting-suspicious	11	14	0	0
1B	Altruist-egoist	80	75	2	-2	3O	Others	22	28	0	0
1C	Humble-proud	26	50	-1.7	1.7	4A	Strong-weak	60	87	-0.7	0.7
1D	Respectful-judgmental	14	21	-0.4	0.4	4B	Active-passive^∗^	57	51	2.0	-2.0
1E	Faithful-unfaithful	10	22	-1.4	1.4	4C	Hardworking-lazy	69	69	1.5	-1.5
1F	Sincere-insincere	56	67	0.4	-0.4	4D	Organized-disorganized	50	71	-0.5	0.5
1G	Just-unjust	3	1	1.3	-1.3	4E	Decisive-indecisive	16	16	0.7	-0.7
1H	Responsible-irresponsible	45	53	0.5	-0.5	4F	Flexible-rigid	37	49	-0.1	0.1
1O	Others	7	9	0	0	4G	Thoughtful-shallow	30	16	3	-3
2A	Visceral-rational^∗^	66	53	2.6	-2.6	4H	Mature-immature	38	52	-0.3	0.3
2B	Warm-cold	92	126	-0.5	0.5	4O	Others	49	49	1.3	-1.3
2C	Optimist-pessimist	33	44	-0.1	0.1	5A	Capable-incapable	7	12	-0.6	0.6
2D	Balanced-unbalanced	61	88	-0.7	0.7	5B	Intelligent-dull	32	32	1	-1
2E	Specific	49	73	-0.8	0.8	5C	Cultured-uncultured	36	33	1.4	-1.4
2F	Sexuality	7	3	1.7	-1.7	5D	Focused-unfocused	11	5	2	-2
2O	Others	7	5	1	-1	5E	Creative-not creative	12	12	0.6	-0.6
3A	Extroverted-introverted	122	127	1.8	-1.8	5F	Specific abilities	5	6	0.1	-0.1
3B	Pleasant-unpleasant	38	68	-1.6	1.6	6A	Ideological values	47	43	1.7	-1.7
3C	Direct-devious	15	14	0.9	-0.9	6B	Specific values	62	76	0.3	-0.3
3D	Tolerant-authoritarian	39	43	0.7	-0.7	6O	Others	1	3	-0.8	0.8
3E	Conformist-rebel	38	52	-0.3	0.3	7A	Body appearance^∗^	2	75	-7.3	7.3
3F	Dependent-independent	41	58	-0.5	0.5	7B	Physical malaise	4	5	0	0
3G	Peaceable-aggressive	18	29	-0.7	0.7	7C	Attitude toward body^∗^	17	70	-4.6	4.6
3H	Sympathetic-unsympathetic	28	38	-0.2	0.2	NA	No inter-rater agreement	51	86	-1.5	1.5


**f*, Frequency of constructs; SR, standardized residuals; NC, non clinical sample; BN, Bulimia Nervosa patients. ^∗^Categories in which frequency value is significantly different from the expected frequency.*

The differences between congruent and discrepant constructs involved in IDs were analyzed separately for both groups. Significant differences were found both for the non-clinical group, χ^2^(7,*n* = 1210) = 36.602, *p* < 0.001, φ = 0.174 and for the clinical group, χ^2^(7,*n* = 1379) = 71.191, *p* < 0.001, φ = 0.227.

Analyzing the standardized residuals, we found that for the non-clinical group, congruent constructs are predominantly moral; while discrepant constructs are mostly personal and emotional (see **Table 4**).

**Table 4:** Frequency and standardized residuals of congruent and discrepant constructs from the non-clinical sample.

		Moral^∗^	Emotional^∗^	Relational	Personal^∗^	Intellectual	Values	Physical
Congruent	*f*	207	192	248	273	76	80	16
	*f_e_*	190.39	203.08	241.16	290.12	74.34	77.97	15.41
	SR	4.33	-2.82	1.63	-3.83	0.65	0.78	0.49
Discrepant	*f*	3	32	18	47	6	6	1
	*f_e_*	19.61	20.92	24.84	29.88	7.66	8.03	1.59
	SR	-4.33	2.82	-1.63	3.83	-0.65	-0.78	-0.49
Total	*f*	210	224	266	320	82	86	17
	*f_e_*	210	224	266	320	82	86	17


*^∗^Areas in which frequency value is significantly different from the expected frequency.*

For BN patients, as shown in **Table 5**, moral constructs and those regarding values and interests were overrepresented as congruent constructs, while personal and physical constructs were more prominent in discrepant constructs.

**Table 5:** Frequency and standardized residuals of congruent and discrepant constructs from the clinical sample.

		Moral^∗^	Emotional	Relational	Personal^∗^	Intellectual	Values^∗^	Physical^∗^
Congruent	*f*	196	181	235	191	60	76	33
	*f_e_*	164.57	174.46	228.14	233.08	59.33	64.27	48.03
	SR	4.96	1.01	0.96	-5.83	0.17	2.79	-4.10
Discrepant	*f*	37	66	88	139	24	15	35
	*f_e_*	68.43	72.54	94.86	96.92	24.67	26.73	19.97
	SR	-4.96	-1.01	-0.96	5.83	-0.17	-2.79	4.10
Total	*f*	233	247	323	330	84	91	68
	*f_e_*	233	247	323	330	84	91	68


*^∗^Areas in which frequency value is significantly different from the expected frequency.*

## Discussion

The findings of this study suggest that the construction pattern, in terms of content, is the difference between patients with BN and women without EDs.

From many theoretical perspectives, it is understood that the excessive focus on the body as the primary source of self-definition may be a compensation, given the lack of other positive attributes perceived by patients with ED (Bruch, 1978; Jacobi et al., 2004; Stein et al., 2013). Despite this relative transtheoretical agreement, there are not many instruments available for systematic exploration of the construction of the self, based on personal meanings. This study showed promising results related to the use of RGT and the CSPC as means to explore the constructions of self and others.

Compared to controls, individuals with BN used fewer constructions from the personal area and many constructs from the physical area, particularly regarding body appearance and attitude toward the body, to construe themselves and others and to anticipate experience. Therefore, it is possible that, due to a lack of constructs to elaborate personal characteristics of self and others, women suffering from BN tend to exacerbate the attention paid to the body, perhaps as an attempt to achieve more predictability and control in social relationships.

When applied to oneself, moral constructions tend to be congruent, while constructs pertaining to the personal area are mostly discrepant, for both clinical and non-clinical participants. This finding suggests that, independently suffering from BN or not, these women perceived themselves as morally acceptable, and they would like to change some characteristics related to personality, character or way of being. Beside these similarities, we found that controls were unsatisfied with their emotionality while patients with BN wanted to change their physical characteristics and their attitude toward their bodies, and some personal characteristics as well. Moreover, results suggest that, due to IDs, change in those physical and personal constructs, although desirable, is linked to an undesirable change in moral constructs and values. This could contribute to the understanding of the difficulty of change in patients. EDs have been considered in both literature and research as ego-syntonic disorders, in which changing may be both desirable and undesirable, since it could be related to personal values (Serpell et al., 1999; Mulkerrin et al., 2016). An understanding about the relationship between a desired change, and the aspects of the self that are congruent and possibly nuclear, may facilitate treatment. Clinicians could benefit from identifying and elaborating IDs with the patient, maintaining those moral constructs and values that are desirable to the patient, while enhancing change.

There are some limitations that need to be acknowledged and taken into account when considering the findings from this study. Due to the low rates of men affected from eating disorders, only women were included in the study. In addition, all women included in the non-clinical sample were students, and the education level of the clinical sample was not recorded or considered for the analysis. Therefore, even though the results were statistically significant, they should be viewed as preliminary. Although requiring further exploration in a large-scale investigation, the findings of this study suggest that the content of personal constructs is relevant for the understanding of disordered eating. An additional research is needed to explore the construct system and its content in patients with other eating disorder diagnoses.

Considering that a weight-based evaluation of the self could be considered as a maintaining factor for eating disorders and a predictor of relapse (Trottier et al., 2013), psychological treatment should aim to elaborate diversified constructions of the self, which may include more aspects from other areas. The findings from this study suggest that RGT and CSPC can be used by clinicians to explore the content of personal constructs used by patients with BN, assessing those areas in which constructs are over-represented and those in which more elaboration is needed.

It is important for clinicians to explore the content of constructs related to symptomatic areas, which could be hindering change, and focus on them to facilitate improvement. The present study must be considered as a limited first step to identify prototypic types of dilemmas for eating disorders. Therapy manuals or protocols could be designed to work with dilemmas in this specific clinical population, as in the ones proposed for social anxiety (Winter, 1998) and depression (Feixas and Compañ, 2015).

## Ethics Statement

The objectives and procedures of this study were discussed with those responsible within the treatment facility in which participants were recruited. Once potential participants were identified, they were informed about the study, emphasizing that their participation was completely voluntary, the assessment would not imply any changes in the treatment, and that they could withdraw from the study asking for the complete deletion of their data at any time before the publication of study. If the participants agreed, they proceeded to sign the informed consent before the interview. Till date, none of the participants has requested the deletion of their data.

## Author Contributions

All authors of this study are part of a research team led by GF. This work is not an isolated study, but arises from the continuing work of this team in the field of eating disorders, to answer questions and limitations identified in previous studies (e.g., Feixas et al., 2010; Dada et al., 2012). The authors have worked together throughout the process, from conceptualization and development of the research problem, contact with treatment centers, data collection, analysis, to the drafting, revision and final approval of the article.

## Conflict of Interest Statement

The authors declare that the research was conducted in the absence of any commercial or financial relationships that could be construed as a potential conflict of interest.

## Footnotes

1 www.usal.es/tcp
